# Book Reviews

**Published:** 1908-06

**Authors:** 


### BOOK REVIEWS

---

THE AMERICAN POCKET MEDICAL DICTIONARY.—Edited by W L. Newman Dorland, M. D., editor "The American Illustrated Medical Dictionary. Fifth Revised Edition. 32mo of 574 pages. Philadelphia and London: W. B. Saunders Company, 1906. Flexible Morocco, gold edges, $1.00 net; thumb indexed, $1.25 net.

W. B. Saunders Company, Philadelphia and London.

This handy little volume contains some thirty thousand medical terms with brief, but clear and adequate definitions, with a considerable amount of matter in tabular form. The vocabulary is well selected and up to date.

A TEXT- BOOK OF PATHOLOGY.—By Alfred Stengel, M. D., Professor of Clinical Medicine in the University of Pennsylvania. Fifth Revised Edition. Octavo of 977 pages, with 399 text-illustrations, many in colors, and 7 full-page colored plates Philadelphia and London; W. B. Sanders Company, 1906. Cloth, $5.00 net; Half Horocco, $6.00 net.

W. B. Saunders Company, Philadelphia and London.

The favorable reception of previous editions has convinced the author that his purpose of supplying a moderate-sized book on clinical pathology has found favor with the profession. In this edition the section dealing with General Pathology has been most extensively revised, several of the important chapters having been practically rewritten. A practical addition is an Appendix treating of the technic of pathologic methods, giving the most importont methods at present in use. The work will be found to present the latest knowledge on Pathology.

---

NERVOUS AND MENTAL DISEASES.—For the Student and Practitioner. By Charles S. Potts, M. D., Professor of Neurology in the Medico Chirurgical College of Philadelphia, Neurologist to the Philadelphia Hospital, etc. Second Revised Edition, enlarged, illustrated, with 133 engravings and 9 plates. Lea & Febiger Publishers, Philadelphia, Pa.

This book comprises 570 pages and presents in rather a concise manner the essential facts of neurology and well fulfills the design of the author in presenting the subject *practically*. We think it advisable that students should begin their study of the various branches of medicine by the use of rather elementary works than in the bewildering large volumes written by specialists and for specialists or practitioners of experience.

Potts has covered the subject in a systematic manner and has incorporated the recent advances in this field of medicine.

The illustrations are good and the binding of the same excellent which characterizes the publications of Lea & Febiger.

GYNECOLOGY AND ABDOMINAL SURGERY.—In two large octavos. Edited by Howard A. Kelly, M. D., Professor of Gynecologic Surgery at Johns Hopkins University; and Charles P. Noble, M. D., Clinical professor of Gynecology at the Woman's Medical College, Philadelphia. Large octavo volume of 851 pages, with 405 original illustrations by Mr. Hermann Becker and Mr. Max Brodel. Philadelphia and London: W. B. Saunders Company, 1907. Per volume, Cloth, $8.00 net; Half Morocco, $9.50 net.

W. B. Saunders Company, Philadelphia and London.

In view of the intimate association of gynecology with abdominal surgery the editors have combined these two important subjects in one work. For this reason the work is doubly valuable, for not only the gynecologist and general practitioner will find it an exhaustive treatise, but the surgeon also will find here the latest technic of the various abdominal operations. It possesses a number of valuable features not to be found in any other publication covering the same fields. It contains a chapter upon the bacteriology and one upon the pathology of gynecology, dealing fully with the scientific basis of gynecology. In no other work can this information, prepared by specialists, be found as separate chapters. There is a large chapter devoted entirely to medical gynecology, written especially for the physician engaged in general practice. Heretofore the genral practitioner was compelled to search through an entire work in order to obtain the information desired. This work presents it all in one chapter. Abdominal surgery proper, as distinct from gynecology, is fully treated. The chapter on Complications of operations will be of great service to every operator, as it considers in detail every complication following operations which can occur. There are also elaborate chapters devoted to Operations during pregnancy, operations before puberty and conservative operations upon the appendages—subjects usually treated of only in monographs. Special attention has been given to modern technic and illustrations of the very highest order have been used to make clear the various steps of the operations. Indeed the six hundred and fifty original illustrations are truly magnificent, being the work of Mr. Hermann Becker, Mr. Max Brodel, and other eminent artists of the Johns Hopkins Hospital.

A TEXT-BOOK OF EMBRYOLOGY.—By John C. Heisler, M. D., Professor of Anatomy in the Medico-Chirurigal College of Philadelphia. Third Revised Edition. Octavo volume of 432 pages, with 212 illustrations, 32 of them in colors. Philadelphia and London: W. B. Saunders Company, 1907. Cloth, $3.00 net; Half Morocco, $4.25 net.
W. B. Saunders Company, Philadelphia and London.

The new edition of this work, just issued, represents all the latest advances recently made in the science of embryology. Many portions have been entirely rewritten and a great deal of new and important matter added. A number of new illustrations have also been introduced and will prove most valuable. The previous editions of this work filled a gap most admirably, and this new edition will undoubtedly prove even more valuable. Heisler's Embryology has become a standard work.

---

CHEMICAL PATHOLOGY.—Being a Discussion of General Pathology from the Standpoint of the Chemical Processes Involved. By H. Gideon Wells, Ph., D., M. D., Assistant Professor of Pathology in the University of Chicago and in Rush Medical College, Chicago. Octavo of 549 pages. Philadelphia and London: W. B. Saunders Company, 1907. Cloth, $3.25 net.
W. B. Saunders Company, Philadelphia and London.

As the chemistry of disease processes is the foundation of practical treatment, the subject is of the utmost importance to the practitioner and the clinical investigator. Dr. Wells here presents the latest work systematically considering the subject of general pathology from the standpoint of the chemical processes involved. It is written for the physician, for those engaged in research in pathology and physiologic chemistry, and for the medical student. In the introductory chapter are discussed the chemistry and physics of the animal cell, giving the essential facts of the theories of the composition of proteids, and of ionization, diffusion, osmotic pressure, etc., and the relation of these facts to cellular activities. Special chapters are devoted to Diabetes and to Uric-acid Metabolism and Gout.

